# Association of *bla_OXA-23_* and *bap* with the persistence of *Acinetobacter baumannii* within a major healthcare system

**DOI:** 10.3389/fmicb.2015.00182

**Published:** 2015-03-12

**Authors:** Ting L. Luo, Alexander H. Rickard, Usha Srinivasan, Keith S. Kaye, Betsy Foxman

**Affiliations:** ^1^Department of Epidemiology, School of Public Health, University of MichiganAnn Arbor, MI, USA; ^2^Division of Infectious Diseases, Wayne State UniversityDetroit, MI, USA

**Keywords:** molecular epidemiology, REP-PCR, *bap*, OXA-23, antibiotic resistance, biofilms

## Abstract

**Objectives:** *Acinetobacter baumannii* is an emerging opportunistic nosocomial pathogen. Two factors that may enhance persistence in healthcare settings are antimicrobial resistance and biofilm-forming ability. The aim of this work was to determine whether *A. baumannii* isolates that persist in healthcare settings (endemic), can be differentiated from sporadic isolates based upon their ability to resist antibiotics and their biofilm-forming capability.

**Methods:** Two hundred and ninety *A. baumannii* isolates were isolated over 17 months in the Detroit Medical Center (DMC). The isolates were genotyped using repetitive extragenic palindromic-PCR (REP-PCR). REP-types appearing greater than 10 times during active surveillance were considered endemic. The *in vitro* biofilm-forming ability and antibiotic resistance profile of each isolate were evaluated. Isolates were tested for the presence of two genetic markers—one implicated in biofilm formation (*bap*) and the other in antibiotic resistance (*bla_OXA-23_)*.

**Results:** Of the 290 isolates evaluated, 84% carried *bap* and 36% carried *bla_OXA-23_*. Five unique REP-PCR banding-types were detected >10 times (endemic) and constituted 58% of the 290 isolates. These five endemic REP-PCR types were 5.1 times more likely than sporadic isolates to carry both *bap* and *bla_OXA-23_*. Furthermore, endemic isolates were resistant to 3 more antibiotic classes, on average, than sporadic isolates and four of the five endemic REP-PCR types formed denser biofilms *in vitro* than sporadic isolates.

**Conclusions:** Endemic *A. baumannii* isolates are more likely than sporadic isolates to possess factors that increase virulence and enhance survival within a large healthcare system.

## Introduction

*Acinetobacter baumannii* is a Gram-negative coccobacillary bacterium that is environmentally ubiquitous. It is capable of causing severe nosocomial infections, especially among patients who are immunocompromised (due to underlying medical conditions or age), have medical devices, or are frequently exposed to antibiotics (Bergogne-Berezin, 2001; Maragakis and Perl, 2008). *A. baumannii* is intrinsically tolerant to many antimicrobials and is capable of rapidly acquiring antibiotic resistances. Consequently, it is not unusual to observe considerable and unexpected increases in the number of resistant isolates within a healthcare facility over short periods of time. For example, within tertiary care facilities in Greece, there were no reported imipenem-resistant *A. baumannii* before 1996, but by 2006 91% of *A. baumannii* that were isolated were imipenem-resistant (Falagas et al., 2007). A similar trend was observed at the Detroit Medical Center (DMC) in Michigan where imipenem-resistant *A. baumannii* isolates increased from 1% to 58% between 2003 and 2008 (Reddy et al., 2010).

Given the volume and spectrum of antimicrobials used in healthcare settings, sometimes to an excessive and inappropriate degree (Craig et al., 1978; Huttner et al., 2013), resistant clonal pathogens can emerge and circulate globally (Butaye et al., 2006; Samuelsen et al., 2011; Martins et al., 2015). Knowledge of the biology behind recirculation and persistence may aid in future prevention and/or treatment strategies. For instance, multidrug efflux pumps that enable the survival of bacteria in the face of antimicrobial challenge have also been suggested to directly influence colonization and persistence (Piddock, 2006). Only a few small-scale studies have investigated the association of antibiotic resistance to the circulation and/or persistence of PCR-genotyped *A. baumannii* isolates in a healthcare system. In Spain, PCR fingerprinting of 29 imipenem-resistant *A. baumannii* isolates collected at a large hospital in Madrid showed that they belonged to a single circulating clone (Bou et al., 2000). A similar study conducted in China found that 24 multi-drug resistant isolates collected from various sources in Sichuan Province had identical REP-PCR patterns (Luo et al., 2011). A study of 23 epidemic *A. baumannii* isolates compared to 23 sporadic isolates collected from 9 European countries demonstrated epidemic isolates were significantly more resistant than sporadic isolates (Koeleman et al., 2001).

An important virulence biomarker in *A. baumannii* is the *bla_OXA−23_* gene, which codes for the carbapenemase OXA-23, one of five Ambler class D β-lactamases identified in *A. baumannii* (Opazo et al., 2012). Outbreaks of OXA-23 producing *A. baumannii* have been characterized worldwide and associated with multi-drug resistance (Coelho et al., 2006). Although OXA-23 production directly confers resistance to carbapenems, possessing the gene is often a marker for other resistances, including cephalosporins, beta-lactams, fluoroquinolones, and aminoglycosides (Dalla-Costa et al., 2003; Girlich et al., 2010). These drug classes are normally used to classify multi-drug resistant *A. baumannii* (Falagas et al., 2006). OXA-23 producers were first isolated in the United States in 2008, from a multidrug resistant *A. baumannii* outbreak in Detroit (Douglass et al., 2008). However, it is unknown whether the presence of the *bla_OXA−23_* gene in *A. baumannii* is associated with persistence.

Biofilms enhance the persistence of microorganisms within adverse environments, serve as a reservoir for dissemination of microorganisms in favorable conditions, and act as a nidus for the exchange of antimicrobial resistance genes (Hall-Stoodley and Stoodley, 2005). Biofilms are surface-associated communities of aggregated microorganisms that produce large amounts of extra-cellular polymeric substances (Donlan and Costerton, 2002). Increasing evidence suggests that the ability of *A. baumannii* to form biofilms enhances antibiotic resistance/tolerance and also promotes persistence in hospital settings (Rao et al., 2008; Rodriguez-Bano et al., 2008). In particular, the biofilm associated protein (*bap*) gene present on the chromosome in some (but notably not all) strains of *A. baumannii* codes for cell-surface-associated Bap that is responsible for the development of three-dimensional biofilm towers and channels on abiotic and biotic surfaces (Brossard and Campagnari, 2012). Transposon mutagenesis of the coding region of *bap* has been shown to reduce the volume and thickness of *A. baumannii* 14 h biofilms by more than 2-fold (Loehfelm et al., 2008). We are aware of only one study that examined the prevalence of the *bap* gene in clinical *A. baumannii* isolates. In a study focusing on clinical *A. baumannii* isolated over a span of 10 years from Royal Brisbane and Women's hospital, the *bap* gene was present in 22 of 24 isolates that were tested (92%) (Goh et al., 2013).

There has not been a large-scale assessment to determine whether endemic healthcare isolates are more likely than sporadic isolates to possess a greater number of antibiotic resistances and enhanced biofilm-forming abilities. For the purposes of this study, identical repetitive extragenic palindromic PCR (REP-PCR) types shared by at least 10 isolates across our study population were considered endemic. The aim of our study was to analyze the prevalence of antibiotic resistance, quantify biofilm formation, and probe for the presence of *bap* and *bla_OXA−23_* among a collection of *A. baumannii* isolates at a large healthcare system in Southeast Michigan. Ultimately, through laboratory-based approaches using diagnostic tests, molecular analyses, and microscopic imaging, we show data that indicates antimicrobial resistance and biofilm formation are linked to circulation and persistence of *A. baumannii*.

## Materials and methods

### Surveillance site

The DMC is a large tertiary referral healthcare system that services Southeast Michigan. It consists of 8 hospitals and over 2000 inpatient beds. The central microbiology lab processes over 500,000 specimens annually. From January 2010 to May 2011, a total of 426 clinical *A. baumannii* isolates were collected from DMC hospitals and facilities. 136 of these isolates were cultured from patients residing at a nursing home that is physically located within DMC. Since our focus is on nosocomial spread, we excluded these isolates. Thus, the final sample size was 290 isolates collected from 7 hospitals that comprise the DMC. Collection of the isolates and their examination was approved by the appropriate Institutional Review Boards at the DMC [059112MP2E] and the University of Michigan [HUM00064166].

### Strains and culture conditions

*A. baumannii* isolates were transferred to the University of Michigan School of Public Health using stab cultures. The stab culture vials were plated onto Bacto™ R2A agar (Reasoner and Geldreich, 1985) and checked for purity by examining colony color and morphology. A colony from each isolate was then picked and inoculated into a set of 1.5 mL cryogenic vials containing R2A broth. The vials were incubated overnight and a frozen working stock (−80°C) was prepared by adding glycerol to a total concentration of 25%. All subsequent re-culturing was performed by sub-culturing a loop-full of the working stock on to R2A agar, minimizing repeated culturing steps. Colonies that were developed from overnight incubation at 37°C were used for comparative studies.

### DNA-template preparation

From a fresh overnight isolation plate on R2A agar, 4–5 colonies were removed using a sterile toothpick and inoculated into a 0.2 mL PCR tube containing nuclease free water. The tubes were vortexed for approximately 30 s to suspend the cells. After suspension, the tubes were boiled at 95°C for 15 min in a Bio-Rad S1000™ Thermal Cycler (Hercules, CA) to lyse the cells as described by Snelling et al. (1996) After boiling, the samples were frozen in −80°C freezers during PCR reagent preparation to enhance lysis of any remaining intact cells.

### Repetitive element palindromic (REP)-PCR genotyping

REP typing was performed using previously published primer sets (Vila et al., 1996). The oligonucleotide primers were prepared by Invitrogen (Carlsbad, CA). Amplification reactions were performed using Promega's GoTaq® Green Master Mix (Madison, WI). Each reaction contained 12.5 μL of GoTaq® Green, 2.5 μL of template, 2.5 μL of 1 μM primer mixture of both forward and reverse primers, 2.5 μL of dimethyl sulfoxide as a stabilizer for GC-rich regions of *A. baumannii* (Ralser et al., 2006), and 5 μL of nuclease-free water. The optimized PCR protocol was as follows: an initial denaturation step of 95°C for 3 min followed by 30 cycles of denaturation at 90°C for 30 s, annealing at 40°C for 1 min, and extension at 65°C for 8 min. A final extension time of 16 min at 65°C was included at the end of the cycle. Amplified products were imaged under UV (300 nm wavelength) and digital images captured. A 1 Kb + ladder was used every 4 lanes to ensure comparability between lanes. Phylogenetic trees were constructed using PyElph version 1.4 (Pavel and Vasile, 2012). Lanes and bands were manually detected and compared to a 1 kb plus ladder lane. The neighbor joining method was used to visually display REP-clusters.

### Definitions of sporadic and endemic

We defined endemic *A. baumannii* as isolates with identical REP-PCR fingerprinting present in counts of greater than 10 across our study population. All other isolates were considered sporadic.

### Detection of *bap* and *bla_OXA−23_*-like genes

For *bla_OXA−23_* we used primer sets as described by Jeon et al. (2005) with modified PCR conditions. The primer set used for *bap* were designed using BLAST by Christine Greene (University of Michigan Department of Environmental Health Sciences). These were bap-F 5′-AGTTAAAGAAGGGCAAGAAG-3′ (forward) and bap-R 5′-GGAGCACCACCTAACTGA-3′ (reverse). The PCR protocols for amplication of *bla_OXA−23_* and *bap* were identical asides from the annealing temperature. The protocols are as follows: 95°C for 1 min as an initial denaturation step followed by 30 cycles of 95°C for 30 s, 49°C/45°C for 30 s, and 72°C for 1 min. A final elongation step at 72°C for 5 min concluded the protocol. The annealing temperature was optimized at 49°C for *bla_OXA−23_* and 45°C for *bap*. Products from the reaction were run on 1% agarose gels at 100 V and visualized under UV (300 nm wavelength) to determine presence or absence of gene.

### Antibiotic susceptibility testing

Antibiotic susceptibility testing was conducted at the clinical laboratory at the Henry Ford Hospital using the broth micro-dilution technique MicroScan (Siemens, Erlangen, Germany). The panel also positively identified the species as *A. baumannii*. Resistant MIC breakpoints were used according to CLSI standards with “1” indicating full resistance (CLSI, 2011). Intermediately susceptible isolates were considered susceptible. There were 16 antibiotics (shown in **Table 2**) in the panel for which there exist 2011 CLSI guidelines. For Tigecycline we used the 2011 BSAC guidelines to determine resistant breakpoints (BSAC, 2011).

### Biofilm quantification

Biofilm quantification was performed on both sterile polystyrene-bottomed and glass-bottomed 96-well plates using a modified crystal violet assay method (O'Toole and Kolter, 1998). Each well was filled with 100 μL of R2A broth and subsequently inoculated with an isolate from the study sample. Isolates were inoculated in triplicate and incubated at 37°C for 18 h. Subsequently, the excess culture media was aspirated and the biofilms were stained with 50 μL of crystal violet. After 15 min of staining, the wells were washed 3× with deionized water for 10 min each wash. After the last wash, the plates were air dried for 30 min and 50 μL of 95% ethanol was added to each well and gently agitated to bring any remaining crystal violet into solution. The O.D. at 560 nm for each triplicate was recorded in a Wallac Victor 3 multilabel plate reader (Perkin-Elmer, Waltham, MA) and the average value calculated.

### Biofilm imaging

Three representative isolates were chosen for imaging. They included a *bap* alone isolate, a *bap* + *bla_OXA−23_* positive isolate, and a *bap* + *bla_OXA−23_* negative isolate (note, no *bla_OXA−23_* alone were detected in our study) of *A. baumannii* (isolates DMC0256, DMC0420, and DMC0551, respectively). A modified method that was developed by Rao and colleagues was used (Rao et al., 2011). Biofilms were developed overnight at 37°C in polystyrene 4-well chamber slide system (Lab-Tek, Naperville, IL) containing 2 mL of R2A media per well. The media was aspirated from each well and stained with prepared 10 μM solution of Syto-9 stain (Invitrogen, Carlsbad, CA) for 30 min. Subsequently, the biofilms were washed with PBS (pH 7.4) three times. A Leica SPE (Leica, Buffalo Grove, IL) confocal laser scanning microscope (CLSM) was used to examine the stained biofilms. Syto-9 stained biofilms were excited with a 488 nm solid-state laser and fluorescence was captured between 500–550 nM with a 40 × 1.25 NA objective lens. Gain and offset were calibrated using the Leica look-up-table and kept constant for experiments. Following biofilm imaging, biofilms were rendered using IMARIS version 7.3.1 (Bitplane, Zurich, Switzerland) imaging software. Captured renderings were assembled in CORELDRAW v. X4 (Corel, Mountain View, CA).

### Definition of multi-drug resistance (MDR) and Pan drug resistance (PDR)

The definition of MDR and PDR *A. baumannii* is not fixed and it varies by researcher in numerous published works (Manchanda et al., 2010). Here, we defined MDR *A. baumannii* as having resistance to any combination of 3 or more of the five drug classes commonly used to treat Gram negative infections: cephalosporins, carbapenems, β-lactam and β-lactamase inhibitor combinations, fluoroquinolones, and aminoglycosides. Resistance to an antibiotic class was defined as resistance to all drugs representative of that class in our panel. PDR *A. baumannii* was defined as resistance to all five antibiotic classes and resistance to tetracycline and trimethoprim-sulfamethoxazole.

### Statistical analyses

All statistical analyses were performed on SAS® 9.3 software. The continuous outcome of mean 560 nm absorbance between comparison groups were tested using student's *t*-test. To determine whether frequency counts are distributed the same across different populations of *A. baumannii*, a chi-square test of homogeneity was used. Significance threshold was set at α = 0.05.

## Results

### Descriptive epidemiology

*A. baumannii* was isolated from 290 patients receiving acute care in the DMC healthcare system between January 2010 and May 2011 (17 month time period). If multiple isolates were obtained from an individual, one was randomly selected for inclusion. The majority of isolates (88%) were taken from three hospitals. The remaining 12% were isolated from 4 other hospitals. *A. baumannii* found from any anatomic site were included. For analysis, the anatomic sites of isolation were categorized by anatomical system. For example, sputum and bronchial alveolar lavage specimens were defined as respiratory. Respiratory (39%), urinary (20%), wound (17%), and blood (14%) were the source for 90% of isolates. The remaining *A. baumannii* isolates were found in tissue, peritoneal/cerebral-spinal fluids, eye/ear/skin swabs, and specimens indicated as “other.” The mean age of the study cohort was 57 years with a standard deviation of 21 years. The number of male patients was 155 (53%). Of the 290 patients, 72% were African American, 16% white, 1% Hispanic, and <1% Asian; 11% patients had undocumented races or were indicated as “other.” A comprehensive breakdown of patient demographics and isolate descriptives by endemicity is shown in Table 1.

**Table 1:** **Comparison of various patient demographic factors and isolate descriptives of 121 sporadic *A. baumannii* and 169 endemic *A. baumannii*^a^^b^ isolated at the Detroit Medical Center between Jan. 2010–May 2011**.

	***A. baumannii* isolate classification**	**Sporadic (*N* = 121)**	**Endemic (*N* = 169)**					***p*-value^c^**
			**REP-1**	**REP-2**	**REP-3**	**REP-4**	**REP-5**	
	**Count**	**121**	**96**	**27**	**16**	**16**	**14**	
Race	Black	82 (68%)	72 (75%)	22 (81%)	13 (81%)	12 (75%)	8 (57%)	0.17
	White	19 (16%)	14 (15%)	3 (11%)	2 (13%)	3 (19%)	4 (29%)	0.94
	Other	15 (12%)	10 (10%)	2 (8%)	1 (6%)	1 (6%)	2 (14%)	0.43
	Hispanic	4 (3%)	0 (0%)	0 (0%)	0 (0%)	0 (0%)	0 (0%)	N/A^d^
	Asian	1 (1%)	0 (0%)	0 (0%)	0 (0%)	0 (0%)	0 (0%)	N/A^d^
Gender	Male	61 (50%)	47 (49%)	16 (59%)	13 (81%)	9 (56%)	9 (64%)	0.38
Facility	Sinai Grace	41 (34%)	44 (46%)	13 (48%)	6 (38%)	8 (50%)	5 (36%)	0.058
	Detroit Receiving	27 (22%)	30 (31%)	10 (37%)	5 (31%)	4 (25%)	5 (36%)	0.071
	Harper-Hutzel	29 (24%)	17 (18%)	1 (4%)	3 (19%)	2 (13%)	4 (29%)	0.089
	Children's Hospital	15 (12%)	0 (0%)	0 (0%)	0 (0%)	0 (0%)	0 (0%)	N/A^d^
	Huron Valley	3 (2%)	1 (1%)	1 (4%)	0 (0%)	2 (13%)	0 (0%)	N/A^d^
	Karmanos Cancer	3 (2%)	1 (1%)	1 (4%)	2 (13%)	0 (0%)	0 (0%)	N/A^d^
	Rehab. Institute	3 (2%)	3 (3%)	1 (4%)	0 (0%)	0 (0%)	0 (0%)	N/A^d^
Site of	Respiratory	43 (36%)	39 (41%)	14 (52%)	4 (25%)	8 (50%)	5 (36%)	0.31
Isolation	Urine	26 (21%)	20 (21%)	3 (11%)	5 (31%)	2 (13%)	2 (14%)	0.59
	Wound	20 (17%)	15 (16%)	4 (15%)	4 (25%)	3 (19%)	2 (14%)	0.99
	Blood	18 (15%)	12 (13%)	3 (11%)	1 (6%)	2 (13%)	2 (14%)	0.65
	Tissue	8 (7%)	5 (5%)	1 (4%)	0 (0%)	1 (6%)	1 (6%)	0.49
	Fluid	3 (2%)	2 (2%)	1 (4%)	0 (0%)	0 (0%)	0 (0%)	N/A^d^
	Other	0 (0%)	2 (2%)	1 (4%)	1 (6%)	0 (0%)	0 (0%)	N/A^d^
	Eye	2 (2%)	0 (0%)	0 (0%)	1 (6%)	0 (0%)	0 (0%)	N/A^d^
	Ear	1 (1%)	0 (0%)	0 (0%)	0 (0%)	0 (0%)	0 (0%)	N/A^d^
	Skin	0 (0%)	1 (1%)	0 (0%)	0 (0%)	0 (0%)	0 (0%)	N/A^d^
Isolate	*bla_OXA-23_*	20 (17%)	58 (60%)	16 (59%)	7 (44%)	2 (13%)	2 (14%)	<0.01
Biomarker	*bap*	93 (77%)	91 (95%)	25 (93%)	15 (94%)	14 (88%)	5 (36%)	<0.01

a *Endemic A. baumannii defined as occurrence of identical REP-PCR patterns in counts greater than or equal to 10 during the course of surveillance. All other REP-types are considered sporadic*.

b *Percentages are calculated with column total in denominator and may not add up perfectly to 100% due to rounding to the nearest whole percent*.

c *p-value is calculated between the endemic (n = 169) group and sporadic (n = 121) group*.

d *Chi-square a weak and non-applicable test due to expected value count <5*.

### REP clusters

**Figure 1:**
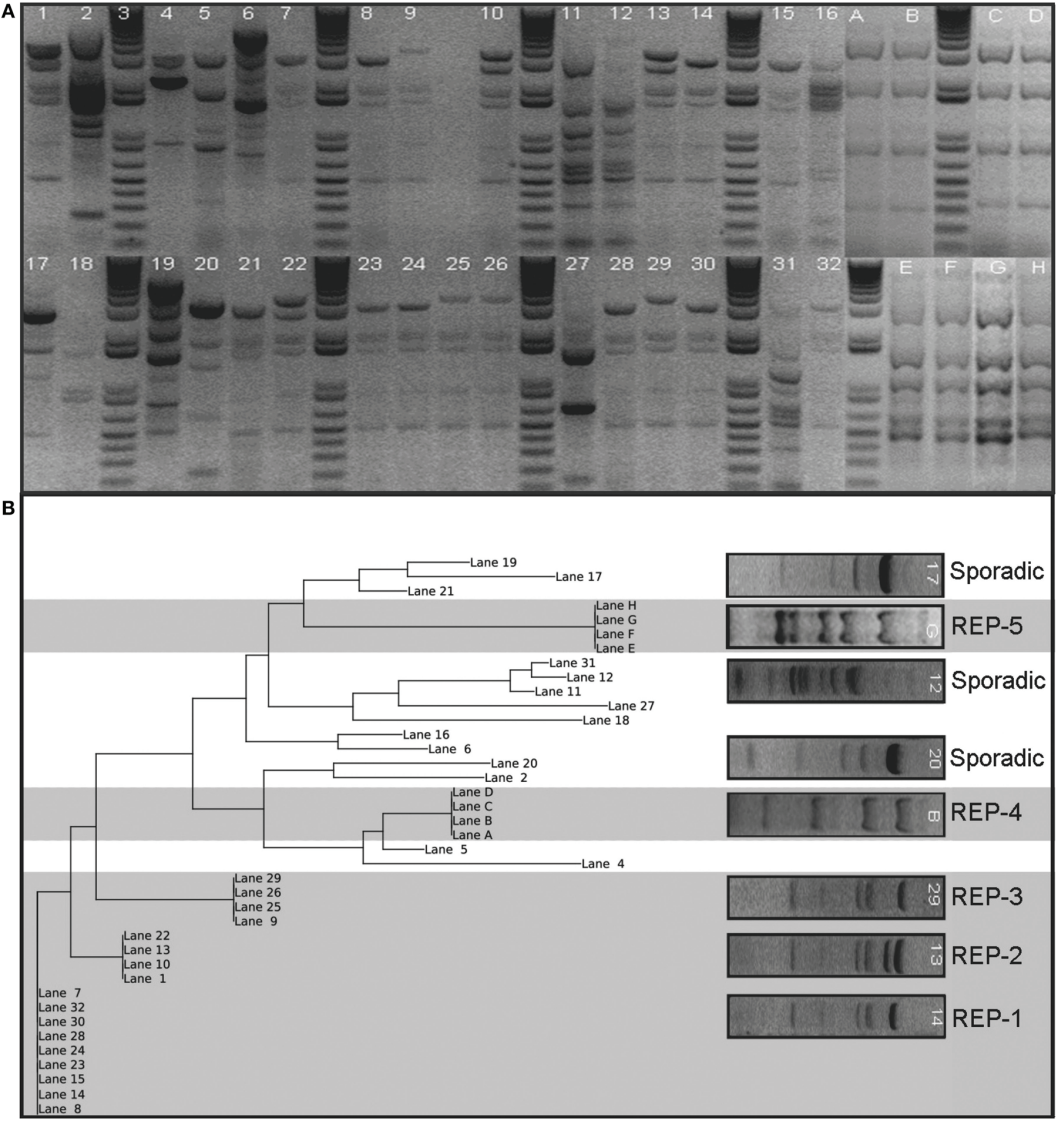
**Sample gel image of the fingerprint generated from REP-PCR of *Acinetobacter baumannii* isolates collected at a single hospital in the Detroit Medical Center system organized by date of isolation**. Panel **(A)** shows lanes 7, 8, 14, 15, 23, 24, 28, 30, and 32 represent REP-1, the most diffusive clone identified. REP-2 and REP-3 are also present on this gel shown by lanes 1, 10, 13, and 22, and lanes 9, 25, 26, and 29, respectively. REP-4 and REP-5 were not run in this sample gel, but are cropped in for comparison. REP-4 is marked Lanes A–D and REP-5 is marked Lanes E–H. Panel **(B)** shows a phylogenetic tree of sample gel using neighbor joining method is also shown. Five clusters are evident and were classified as REP clones. The clusters are indicated by grayed background. The others were designated sporadic isolations and are indicated by a white background. Lettered lanes were added to the analysis to include REP-4 and REP-5 and were derived from separate gels from numbered lanes.

**Figure 2:**
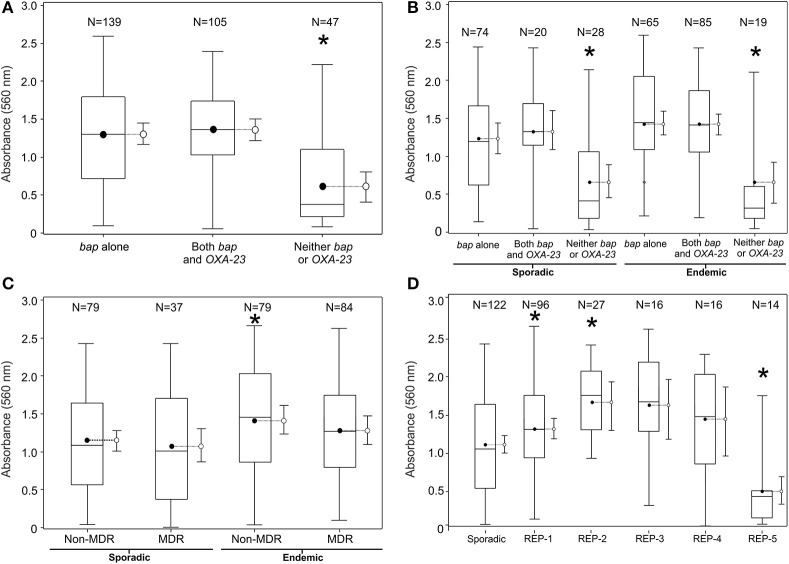
**Biofilm quantification by various groupings of the population of 290 clinical *A. baumannii* isolates collected using active surveillance at the Detroit Medical Center between Jan. 2010-May 2011**. The referent boxplot for each panel is the panel on the far left. A ^*^ indicates significant difference in mean 560 nm absorbance from referent boxplot (*p* < 0.05). **(A)** Breaks down the population of isolates by genetic biomarker, **(B)** by clonality and MDR phenotype, **(C)** by REP-type, and **(D)** by clonality and genetic biomarker. 95% confidence limits are also included to the right side of each box plot.

There were a total of 98 REP genotypes identified amongst the 290 *A. baumannii* isolates tested, 5 of which were observed at least 10 times in the collection. REP-1 and REP-2 were observed in 6/7 hospitals; REP-3 and REP-4 were observed in 4/7 hospitals, and REP-5 was observed in 3/7 hospitals. One hospital, Children's Hospital, had no endemic REP-type isolations. REP-1 and REP-2 occurred at least 10 times in a single hospital. One genotype, REP-1, accounted for 33% of isolates (*n* = 96). Figure 1 shows a sample gel image (Figure 1A), and associated cladogram (Figure 1B). The remaining four predominant REP genotypes were 9% (REP-2), 6% (REP-3), 6% (REP-4), and 5% (REP-5) of the total (Figures 1B, 2D). The next highest REP genotype occurred among only 2% of the isolates. This genotype together with the remainder of the REP-genotypes was considered to be sporadic. REP-1 through REP-5 will be referred to as endemic isolates in the remainder of the manuscript. Days between isolation of the same REP-type within a single hospital ranged from 0 to 203 days (average = 29 days). REP-1 predominated: at-least one isolate was identified in every month of surveillance. REP-types were associated with specific facilities (chi-square *p* = 0.01). Seventy-four of 96 (77%) REP-1 isolates were isolated from just two hospitals. A third hospital had only sporadic REP-types. Endemic isolates were not associated with anatomic site of isolation (*p* = 0.96).

### Presence of *bap* and *bla_OXA−23_* genes

243 (84%) isolates in the study possessed the *A. baumannii bap* gene and 105 (36%) possessed the *bla_OXA−23_* gene. Of the 243 that had *bap*, 138 possessed it alone and the other 105 had the combination of *bap* and *bla_OXA−23_*. No isolates possessed *bla_OXA−23_* independently of *bap*. Endemic isolates were more likely than sporadic to carry *bla_OXA−23_* genes and *bap* genes and these differences are statistically significant (Table 1).

### Antibiotic resistance

MIC determinations were available for all but 6 endemic and 6 sporadic strains. These 12 isolates did not culture adequately for MicroScan tests at Henry Ford Hospital. Endemic isolates were more likely to be resistant (*p* < 0.05) to each antibiotic tested except for amikacin and tigecycline (Table 2), and more likely to be characterized as MDR than sporadic isolates (*p* < 0.05). When observing antibiotic resistance patterns by *bap* and *bla_OXA−23_* genes, isolates with *bap*, but not *bla_OXA−23_* possessed the least number of resistances to the panel of antibiotics (including *A. baumannii* isolates that did not contain either *bap* or *bla_OXA−23_*). Isolates with *bla_OXA−23_* possessed the most number of resistances to the panel of antibiotics. One isolate was resistant to all 17 antibiotics in the panel. This isolate belonged to REP-3, an endemic REP genotype we observed 16 times.

**Table 2:** **Count and percent of *A. baumannii* isolates resistant to selected antibiotics by genetic biomarker and REP-Genotype**.

		**Genetic biomarker**			**REP-Genotype**		**All isolations**
**Group**		**Neither *bap* nor *bla_OXA-23_***	***bap alone***	**Both *bap* and *bla_OXA-23_***	**Sporadic**	**Endemic**	**Total study population**
**Count**		**47**	**131**	**100**	**115**	**163**	**278**
Cephalosporins	Ceftriaxone	72	58	91^^*^^÷^^	60	81^^*^^	72
	Ceftazidime	72	56	91^^*^^÷^^	58	81^^*^^	72
	Cefotaxime	79	60^^*^^	92^^*^^÷^^	62	84^^*^^	75
	Cefepime	74	52^^*^^	91^^*^^÷^^	59	77^^*^^	70
	All 4 cephalosporins	66	45^^*^^	83^^*^^÷^^	50	71^^*^^	62
Carbapenems	Imipenem	40	13^^*^^	75^^*^^÷^^	30	47^^*^^	40
	Meropenem	60	27^^*^^	82^^*^^÷^^	43	58^^*^^	52
	All 2 carbapenems	40	12^^*^^	75^^*^^÷^^	29	47^^*^^	40
B-lactam + inhibitor	Ampicillin + sulbactam	40	21^^*^^	52^^÷^^	22	45^^*^^	36
	Piperacillin + tazobactam	70	34^^*^^	84^^÷^^	47	66^^*^^	58
	Ticarcillin + clavulanate	51	26^^*^^	84^^*^^÷^^	40	59^^*^^	51
	All 3 β-lactams + inhibitors	32	9^^*^^	43^^÷^^	16	32^^*^^	25
Fluoroquinolones	Ciprofloxacin	77	65	96^^*^^÷^^	63	88^^*^^	78
	Levofloxacin	70	59	87^^*^^÷^^	55	82^^*^^	71
	All 2 fluoroquinolones	70	58	87^^*^^÷^^	54	82^^*^^	71
Aminoglycosides	Amikacin	47	22^^*^^	74^^*^^÷^^	38	50	45
	Gentamicin	47	50	72^^*^^÷^^	47	65^^*^^	58
	Tobramycin	23	25	50^^*^^÷^^	23	41^^*^^	34
	All 3 Aminoglycosides	13	7	45^^*^^÷^^	13	28^^*^^	22
Other antibiotics	Tetracycline	40	29	49^^÷^^	28	45^^*^^	38
	Trimethoprim + sulfamethoxazole	66	56	92^^*^^÷^^	57	80^^*^^	71
	Tigecycline^b^	11	5	10	6	9	8
Phenotype	Multi-drug resistant^c^	47	18^^*^^	76^^*^^÷^^	32	52^^*^^	44
	Pan-drug resistant^d^	0	0	7	1	4	3

*Two-hundred and seventy-eight phenotyped clinical A. baumannii isolates collected using active surveillance at the Detroit Medical Center between Jan. 2010–May 2011^ae^*.

*^a^Columns within genetic biomarker and REP-genotype are mutually exclusive, but columns between genetic biomarker and REP-genotype are not*.

b *Resistance determined using BSAC breakpoint*.

c *Multi-drug resistance defined as resistance to all antibiotic representatives in at least three of the five following classes: cephalosporins, carbapenems, β-lactam + inhibitor combinations, fluoroquinolones, and aminoglycosides*.

d *Pan-drug resistance defined as resistance to all antibiotic representatives in all five of the following classes: cephalosporins, carbapenems, β-lactam + inhibitor combinations, fluoroquinolones, and aminoglycosides, in addition to resistance to tetracycline and trimethoprim-sulfamethoxazole*.

e *Isolations include invasive and non-invasive A. baumannii*.

f *Resistance was not available for 12 isolates. See text*.

* *Difference in proportion significant at the 5% level from the baseline group. Baseline for genetic biomarker is neither bap nor OXA-23 and baseline for REP-genotype is sporadic*.

÷ *Difference in proportion significant at the 5% level between bap alone group and bap + OXA-23 group*.

### Biofilm quantification

Biofilm development as determined by biofilm biomass was measured by optical absorbance (560 nm) for all 290 isolates using crystal violet. On hydrophobic polystyrene surfaces the mean absorbance was 1.2 with a standard deviation of 0.7 and a range from <0.1 to 2.6. For relatively hydrophilic glass surfaces, the mean absorbance was 0.9 with a standard deviation of 0.5 and a range from <0.1 to 3.0 (Supplementary Table 1). Irrespective of whether an isolate was grown on glass or polystyrene, a similar trend was observed whereby the highest and lowest biofilm-formers on one surface-type displayed the same biofilm-forming abilities on the other surface-type (Supplemental Figure 1).

Biofilm biomass was significantly greater among endemic and sporadic isolates carrying *bap* (Figures 2A,B), but was not associated with MDR (Figure 2C). Endemic REP types 1–4 generated biofilms of greater average biomass than the average biomass produced by the sporadic isolates, whereas REP type 5 averaged biofilms of lower biomass (Figure 2D). This result is also reflected in the prevalence of the *bap* gene amongst the REP types. Prevalence of *bap* in REP types 1–4 range from 88% to 95%; the prevalence of *bap* in REP type 5 is 36%. Overall, endemic isolates averaged biofilms of greater biomass than sporadic isolates on both polystyrene (hydrophobic) surfaces (1.3 vs. 1.1, *p* < 0.05) and glass (relatively hydrophilic) surfaces (1.0 vs. 0.8, *p* < 0.05).

**Figure 3:**
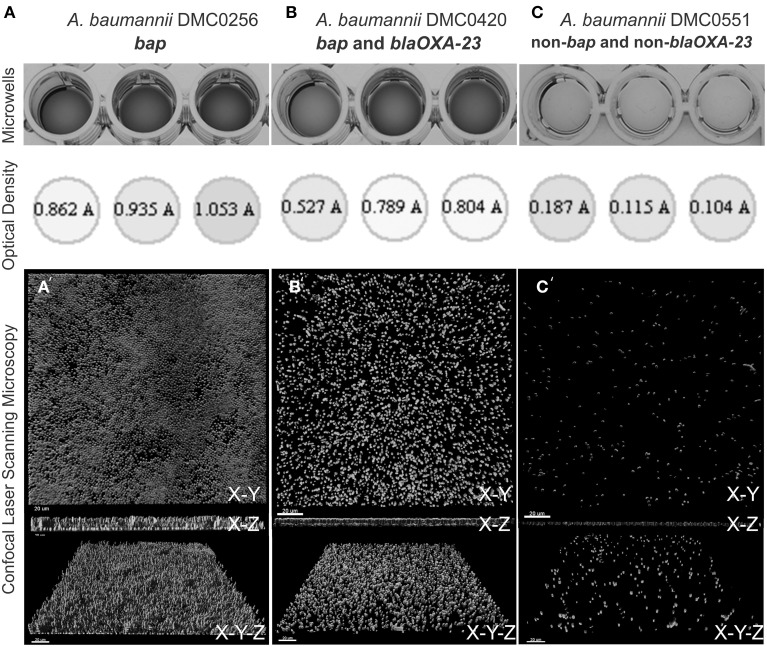
**Biofilm quantification process of four *A. baumannii* isolates using crystal violet staining, measurement from a 96-well plate reader, and visualization using confocal laser scanning microscopy**. **(A)** Shows DMC0256, a representative sample *bap* alone isolate. **(B)** Shows DMC0420 with both *bla_OXA-23_* and *bap*. **(C)** Shows DMC0551, a representative non-*bap* isolate. A top view, side view, and angled view of the biofilm from each isolate is shown to illustrate biomass, thickness, and confluence differences between the isolates.

Microscopic visualization of biofilms of three randomly selected *A. baumannii* isolates that contained the *bap* gene, *bap* and *bla_OXA−23_* genes, or neither of these genes (remembering that no *bla_OXA−23_* alone were detected in our study) showed that there was a clear relationship between the results from the crystal violet assay and the amount of observed biomass (Figure 3). Strains containing *bap* or *bap* and *bla_OXA−23_* were thicker, covered greater surface area, and possessed three-dimensional tower-like structures containing multiple cells compared to strains without *bap* and *bla_OXA−23_*, which formed scant biofilms with most of the biofilm cells being alone or in clusters of 2–5 cells.

## Discussion

In this study of 290 *A. baumannii* isolates conducted using clinical surveillance in a large healthcare system, endemic healthcare isolates were more likely than sporadic isolates to be antibiotic resistant, carry the *bap* and *bla_OXA−23_* genes, and have enhanced biofilm-forming abilities. These findings are exciting as this suggests that the ability to form biofilms may, independent of antibiotic resistance, enhance *A. baumannii's* ability to persist and recirculate within a healthcare system.

We found 5 REP-PCR types that occurred more than 10 times amongst unique patients during the 17 months of surveillance. Furthermore, for 16 of the 17 study months, multiple REP clusters were isolated. This suggests that rather than an outbreak characterized by a rapid dissemination of a single clone, multiple REP types were observed to be recirculating within the DMC. When considering all endemic isolates vs. all sporadic isolates, 52% of all the endemic REP-clones were MDR (vs. 32% for the sporadics), 50% carried *bap* and *bla_OXA−23_* (vs. 17% for the sporadics), and the endemic isolates formed statistically significant denser biofilms than their sporadic counterparts (average of 1.3 vs. 1.1, respectively). These numbers further support our hypothesis that antibiotic resistance markers and biofilm-forming ability are associated with persistence.

Previous studies have estimated the prevalence of *bap* and *bla_OXA−23_* but, unlike this study, most have not associated these genes with antibiotic resistance or biofilm characteristics. A recent study in Australia demonstrated that among 24 isolates of *A. baumannii* from a single outbreak, 22 (92%) possessed the *bap* gene (Goh et al., 2013). Among our surveillance isolates, *bap* was also common, occurring in 84% of the isolates. The biomarker *bla_OXA−23_* was present in 36% of our surveillance isolates, occurred exclusively in the presence of *bap*, and was strongly associated with endemic REP-PCR types and multi-drug resistance. Furthermore, 76% of *bla_OXA−23_* positive isolates were multi-drug resistant and 7% were pan-drug resistant. The percentages are difficult to compare with other studies since most previous studies selected a subset of carbapenem-resistant *A. baumannii* to test for presence of *bla_OXA−23_* rather than an entire clinical population. For example, in Brazil, 13/500 carbapenem-resistant *A. baumannii* isolated from 16 hospitals between 2004 and 2008 were selected for molecular analyses and all 13 isolates were found to be OXA-23 producers (Martins et al., 2009). The *bla_OXA−23_* gene has been detected in MDR outbreaks worldwide (Peleg et al., 2008), but limited studies have implicated OXA-23 producing *A. baumannii* outbreaks in North American hospitals.

Classifications of multi-drug and pan-drug resistance can be vague. This is because the classifications can be based on different combinations of the drug classes commonly used to treat *A. baumannii*. These drugs include cephalosporins, beta-lactam and beta-lactamase inhibitors, carbapenems, fluoroquinolones, aminoglycosides, tetracyclines, and polymyxins (Doi et al., 2015). For the work presented here, surveillance spanned 17 months. This gives us a sustained longitudinal overview of the loss or persistence of antibiotic resistances expressed by the *A. baumannii* population present in the DMC. Of possible concern is that 70–78% of the isolates were resistant to the 4 cephalosporins and the 2 fluoroquinolones in our panel over the 17 month period (Table 2). Carbapenems, often considered the last resort antibiotics for *A. baumannii* (Smith et al., 2013), were ineffective against 40–52% of our isolates. Beta-lactams/beta-lactamase inhibitor combinations and aminoglycosides retained moderate efficacy with 34–58% inefficacy against our collection. The high prevalence of multi-drug resistance has significant public health implications. In some instances, there are limited treatment options for symptomatic patients. Asymptomatic patients carrying *A. baumannii* may also serve as a reservoir and aid in spread to other facilities, which may ultimately prolong the circulation of MDR *A. baumannii* within a facility (Marchaim et al., 2007).

Our data suggest that an increased ability to form biofilms does not confer increased resistance to antibiotics, after controlling for whether isolates were endemic or sporadic (Figure 2C). The absence of an interacting effect is at first-glance surprising given that bacteria in biofilms have altered growth properties, which reduces antimicrobial susceptibility, and many antimicrobials can undergo reaction diffusion limitation when penetrating biofilms (i.e., biofilms of increasing thickness retard access) (Manchanda et al., 2010). However, our approach to test antimicrobial susceptibility was based upon the MicroScan broth microdilution test that relies upon planktonic growth. Therefore, the ability to detect a relationship between biofilm formation and antimicrobial resistance is based upon an environmentally unrepresentative situation. Growth in planktonic phase, as opposed to the *preferred* biofilm growth, could be the reason why isolates carrying *bap* alone were more susceptible to all antibiotics listed in our panel than those without *bap* and those carrying the combination of *bap* and *bla_OXA−23_* (Table 2). Whether this is a causal association or one that is due to the Bap protein enhancing antimicrobial tolerance in biofilms but reducing resistance in planktonic populations remains to be determined. We found no other studies in the literature for comparison.

When considering our approach to evaluate biofilm formation, the crystal violet staining technique is a tried-and-tested technique capable of accurately estimating biofilm formation especially when large panels of isolates are being evaluated (Van Houdt et al., 2004; O'Toole, 2011; Rao et al., 2011; Coffey and Anderson, 2014). We found that the variation of measurements within an isolate were very consistent amongst the triplicate measurements (Figure 3), but between isolates ranged considerably. This is shown by the large standard deviations and wide range of absorbance values in Supplementary Table 1. When considering the variation in biofilm forming ability between the different strains, it is very likely that *bap* is one in a suite of genes that can enhance biofilm development. For example, *A. baumannii* pili is integral for initial attachment to surfaces prior to biofilm maturation (Nait Chabane et al., 2014). Similarly, the products of other genes may change the properties of *A. baumannii* cells, such as whole-cell hydrophobicity and altered bacterial cell-cell signaling that can retard biofilm forming ability (Niu et al., 2008; Pour et al., 2011; Kempf et al., 2012). This may explain why, for example, we observed some *bap* isolates that were poor at forming biofilms and vice-versa.

Some may question the clinical relevance of the observed statistically significant differences in biofilm forming ability between endemic and sporadic isolates as the absolute difference in measures is small. However, given that optical density measurements above approximately 0.5 do not linearly correlate with biomass (poor correspondence with Beer's law) (Lee and Lim, 1980; Fuchs and Kroger, 1999), it is likely that the actual difference in biomass between the average optical densities is much larger than conveyed by the biofilm assay. Further, we observed the same significant differences in biofilm biomass between endemic and sporadic isolates on two contrastingly different surfaces (hydrophobic polystyrene and relatively hydrophilic glass).

In summary, the work presented here indicates that *A. baumannii* isolates endemic to hospitals are more likely than sporadic isolates to be resistant to antibiotics, carry *bap* and *bla_OXA−23_*, and form biofilms of greater biomass *in vitro*. This suggests that specific environmental pressures select for characteristics, such as biofilm formation, that enhance survival of *A. baumannii* in healthcare environments. Taken in context with other studies, our work highlights the importance of antimicrobial stewardship (Fishman, 2006; Garg and Guez, 2011) and the potential application of novel “anti-biofilm” technologies (Simões et al., 2010; Thallinger et al., 2013) to control *A. baumannii* in hospital environments.

### Conflict of interest statement

The authors declare that the research was conducted in the absence of any commercial or financial relationships that could be construed as a potential conflict of interest.
