# Supplementary material for: Association of blaOXA-23 and bap with the persistence of Acinetobacter baumannii within a major healthcare system
Source: Front Microbiol. 2015 Mar 12;6:182. doi: 10.3389/fmicb.2015.00182 (PMC4357298; doi:10.3389/fmicb.2015.00182)
Supplement: Supplementary file 1 [file DataSheet1.DOCX]

**Supplementary Figure 1:** Biofilm quantification of 290 isolates of *A. baumannii* developed on polystyrene and glass as determined by crystal violet staining and absorbance readings at 560 nm. The collection was isolated from the Detroit Medical Center between January 2010 and May 2011. The correlation value indicates moderate and positive degree of association between biofilm formation on glass and biofilm formation on polystyrene.

**
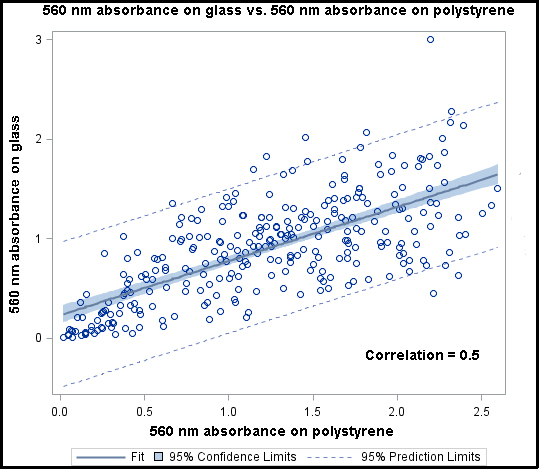
**

| **Supplementary Table 1:** Biofilm quantification represented by crystal violet staining and 560 nm absorbance by genetic biomarker and REP-genotyping.^abc^ 290 *A. baumannii* clinical isolates collected at the Detroit Medical Center between Jan. 2010-May 2011 | | | | | | |
| --- | --- | --- | --- | --- | --- | --- |
| **Polystyrene surface of 96-well microtitre plate** | | | | | | |
|  | **genetic biomarker** | | | **REP-genotype** | | **All Isolations** |
| **Group** | **Neither *bap* nor *bla_OXA-23_*** | ***bap alone*** | **Both *bap* and bla*_OXA-23_*** | **Sporadic** | **Endemic** | **Total study population** |
| **Count** | **47** | **138** | **105** | **121** | **169** | **290** |
| **mean** | 0.6 | 1.3^*^ | 1.4^*^ | 1.1 | 1.3^*^ | 1.2 |
| **std. deviation** | 0.6 | 0.6 | 0.5 | 0.6 | 0.7 | 0.7 |
| **min** | 0.0 | 0.1 | 0.1 | 0.0 | 0.0 | 0.0 |
| **max** | 2.2 | 2.6 | 2.4 | 2.4 | 2.6 | 2.6 |
| **Glass surface of 96-well microtitre plate** | | | | | | |
| **mean** | 0.3 | 0.9^*^ | 1.1^*÷^ | 0.8 | 1.0^*^ | 0.9 |
| **std. deviation** | 0.3 | 0.5 | 0.5 | 0.5 | 0.5 | 0.5 |
| **min** | 0.0 | 0.1 | 0.1 | 0.0 | 0.0 | 0.0 |
| **max** | 1.3 | 2.3 | 3.0 | 2.3 | 3.0 | 3.0 |

^a^ Columns within genetic biomarker and REP-genotype are mutually exclusive, but columns between genetic biomarker and REP-genotype are not
^b^ Isolations include invasive and non-invasive *A. baumannii*.
^c^ Counts different from Table 1 since 12 isolates did not yield MIC results.
^*^ Difference in means significant at the 5% level from baseline group. Baseline for genetic biomarker is neither *bap* nor *bla_OXA-23_*. Baseline for REP-genotype is sporadic.
^÷^ Difference in proportion significant at the 5% level between *bap* alone group and *bap*+ *bla_OXA-23_* group.
